# Multi-kingdom microbiota and functions changes associated with culture mode in genetically improved farmed tilapia (*Oreochromis niloticus*)

**DOI:** 10.3389/fphys.2022.974398

**Published:** 2022-09-12

**Authors:** Haojun Zhu, Jun Qiang, Quanjie Li, Zhijuan Nie, Jiancao Gao, Yi Sun, Gangchun Xu

**Affiliations:** Key Laboratory of Integrated Rice-Fish Farming Ecology, Ministry of Agriculture and Rural Affairs, Freshwater Fisheries Research Center, Chinese Academy of Fishery Sciences, Wuxi, China

**Keywords:** aquaculture, tilapia, in-pond raceway system, gut microbiome, 16S rrna

## Abstract

Genetically improved farmed tilapia (GIFT, *Oreochromis niloticus*) are intensively farmed in China, where most of the yield derives from the pond culture system (PCS). The in-pond raceway system (IPRS) is a new type of highly efficient aquaculture mode, and has been recommended as a novel system for GIFT farming. To determine the effects of these culture modes on the gut microbiome of GIFT, we conducted a 90-days experiment in IPRS and PCS units. A 16S rRNA gene profile analysis showed that the composition of gut microbiota in GIFT under IPRS and PCS conditions gradually separated as rearing progressed, with divergent responses by the midgut and hindgut bacteria. The α-diversity in hindgut decreased significantly by day 90, as compared with on day 7 (*p* < 0.05), with a significantly greater decrease in PCS-reared fish than in IPRS fish (*p* < 0.05). The α-diversity of microbiota in midgut remained stable (*p* > 0.05). The overall dominant gut bacteria were Bacteroidetes, Proteobacteria, and Firmicutes. Rearing mode affected the taxonomic profile of the gut bacteria; in midgut, IPRS samples had more Firmicutes and Fusobacteria compared with PCS samples, but less Proteobacteria, Verrucomicrobia, and Actinobacteria. Firmicutes was enriched in IPRS hindgut, and Fusobacteria was enriched in PCS hindgut. Using random-forest models and LEfSe, we also screened core taxa that could discriminate between the gut microbial communities under IPRS and PCS conditions. The genus *Cetobacterium* (of family Fusobacteriaceae) was significantly enriched in midgut in IPRS fish, and enriched in hindgut in PCS fish. The genus *Clostridium* sensu stricto (of family Clostridiaceae 1) was significantly enriched in both IPRS midgut and hindgut. Analysis with PICRUSt2 software revealed that the culture modes were similar in their effects on the gut microbial metabolic functions. The predicted pathways were significantly enriched in the metabolism class (level 1). Further, the relative abundance of functions related to amino acid metabolic, carbohydrate metabolic, energy metabolic, and metabolic of cofactors and vitamins were high at hierarchy level 2, as the metabolic activity of intestinal bacteria is especially active. Overall, this study enhances our understanding of the characteristics of gut microbiota in GIFT under IPRS and PCS culture modes. Moreover, our findings provide insights into the microecological balance in IPRS units, and a theoretical reference for further development of this culture system.

## 1 Introduction

Since 2000, China has maintained its role as the largest global producer, processor, and trader of fish (Cao et al., 2015). The country has an oversized role in aquaculture production. China alone supplied 57.5 and 59.6% of the global aquaculture volume and value, respectively, for all categories combined in 2020 (Food and Agriculture Organization of the United Nations, 2022). As the main mode of aquaculture in China, the pond culture system (PCS) was estimated to account for about 74% of total freshwater aquaculture production in 2020 (Fishery Administration Bureau of the Ministry of Agriculture and Villages et al., 2021). However, growing problems caused by the traditional, intensive pond culture mode, including the limitations of finite land and water resources, the disruption of aquatic ecosystems, frequent disease outbreaks, the unregulated discharge of wastewater, and the quality and safety of the products, have hindered the development of freshwater aquaculture in China (Zhao et al., 2022). For the problems brought about by PCS, much research has been carried out both in China and abroad (Luau et al., 2020; Song et al., 2020; Couto et al., 2021).

The in-pond raceway system (IPRS) is a new type of high-efficiency farming mode, aiming to better improve water quality and allow increased feeding density, thus increasing production efficiency and profitability when compared with conventional earthen ponds. This culture technology was introduced into China through the U.S. Soybean Export Council in 2013. We modified and upgraded the IPRS to consist of a culture unit occupying 2–5% of the total pond area constructed, and the remaining 95–98% of the pond has a large area to purify the aquaculture tail water (Wang et al., 2019a). The culture unit is used for high-density fish culturing and equipped with water aeration equipment and suction facilities. The purification area is used as a wastewater treatment area to reduce suspended solids and remove organic nutrients (nitrogen and phosphorus) released by cultured fish (Yin et al., 2019a). One major advantage of the IPRS is the ability to manipulate the water velocity and/or exchange rate, thus allowing for the most efficient use of land and water, greater stocking densities, increased production, and better nutrient recycling (Brown et al., 2011). To date, the species that have been reared using the IPRS include tilapia *Oreochromis niloticus* (Yin et al., 2019b), rainbow trout *Oncorhynchus mykiss* (Sirakov et al., 2011), grass carp *Ctenopharyngodon idella* (Zhang et al., 2015), largemouth bass *Micropterus salmoides* (Wang et al., 2020), yellow catfish *Tachysurus sinensis* (Liu et al., 2019), and whiteleg shrimp *Litopenaeus vannamei* (Reid and Arnold, 1992).

Genetically improved farmed tilapia (GIFT, *Oreochromis niloticus*) have many beneficial characteristics including their stable genetic traits, fast growth, high fillet yield, and strong disease resistance (Qiang et al., 2016). Consequently, tilapia is one of the main cultured species in China. Most of the GIFT yield comes from the PCS. Recently, the IPRS was recommended as a novel aquaculture system for GIFT farming. The culture density in IPRS units is more than 10-times that in traditional culture ponds (Wang et al., 2019b). Under the condition of high-density breeding, the intestinal health of the reared animals is particularly important. Many microorganisms inhabit the intestinal tract of animals and play vital roles in maintaining the balance of the intestinal environment and health of the host (Björkstén, 2010). Intestinal microorganisms have an important influence on the host’s metabolism (Valentina and Fredrik, 2012; Bing et al., 2017), growth (Turnbaugh et al., 2009; Zheng et al., 2016), and immunity (Jiao and Wang, 2013; Qi et al., 2017). To date, research on the IPRS technique has mainly focused on the aspects of high yield, system design, and removal of pollutants from the units, while there have been few reports on intestinal microorganisms under IPRS conditions. Therefore, this study used 16S rRNA high-throughput sequencing technology to analyze the intestinal microorganisms of GIFT under IPRS and PCS. The study aimed to evaluate changes in the composition and functions of the gut microbiota of GIFT based on culture mode, by means of a 90-days feeding experiment. Our findings will provide valuable information about the interaction among intestinal microbiota and culture mode. It also provides a reference for screening useful beneficial microbes and regulate intestinal health through microecological agents.

## 2 Materials and methods

### 2.1 Experimental set-up

This experiment was conducted in ponds and in an IPRS, as described by Wang et al. (2019b), at the Yangzhong experimental base of the Freshwater Fisheries Research Center, Chinese Academy of Fishery Sciences, in Jiangsu, China. Healthy juvenile fish were obtained from the Freshwater Fishery Research Center of the Chinese Academy of Fishery Sciences (Yixing, China). Before the experiment, the fish were held for 2 weeks in separate ponds to acclimatize them to the experimental conditions. Juvenile GIFT with an average body weight of 26.45 ± 2.32 g (mean ± SD) were stocked into five 220-m^3^ raceways (20,000 fish per raceway, IPRS) and two 1 500-m^3^ ponds (2,500 per pond, PCS) respectively. Fish were fed a commercial feed, widely used in China, having the following composition: crude protein ≥26%, crude fat ≥4%, crude fiber ≥15%, crude ash ≤18%, water content ≤12%, total phosphorus ≥1%, and lysine ≥1.2%), three times daily, at 7:00 h, 11:30 h, and 16:00 h. The amount of diet was ∼4% of GIFT body weight, and was increased or decreased depending on the residual feed intake the previous day. Culture conditions during the 90-days feeding experiment were: water temperature 23.3–29.8°C, dissolved oxygen 3.41–7.33 mg/L, pH 7.38–8.52, and ammonia-N 0.23–1.86 mg/L. During the whole period of production, there were no changes in the water and no use of aquaculture drugs.

### 2.2 Sample collection

On day 7 (D7) and day 90 (D90) from the start of the experiment, the GIFT were fasted overnight and then harvested, with 10 fish sampled from a given raceway or pond. To avoid the effects of stress on the various measurement indexes, the fish were anesthetized by immersion in 1% MS-222 before being killed. According to anatomical methods, the intestine was divided into foregut, midgut and hindgut, and then midgut and hindgut tissues were collected, frozen in liquid nitrogen, and stored at −80°C until further analysis. A total of 80 intestinal samples were obtained (Table 1).

**TABLE 1:** Distribution of intestine samples from genetically improved farmed tilapia, under two culture modes. RMI = IPRS, midgut, D7; RMF = IPRS, midgut, D90; RHI = IPRS, hindgut, D7; RHF = IPRS, hindgut, D90; TMI = PCS, midgut, D7; TMF = PCS, midgut, D90; THI = PCS, hindgut, D7; THF = PCS, hindgut, D90.

Culture mode	Intestinal segment	Sampling time (d)	Group	*n*
In-pond raceway system (IPRS)	Midgut	7	RMI	10
		90	RMF	10
	Hindgut	7	RHI	10
		90	RHF	10
Pond culture system (PCS)	Midgut	7	TMI	10
		90	TMF	10
	Hindgut	7	THI	10
		90	THF	10

### 2.3 DNA extraction and 16S rRNA gene sequencing

DNA was extracted from the gut samples using an E.Z.N.A.^®^ Stool DNA Kit (D5625, Omega, Inc., United States). The V3-V4 hypervariable region of 16S rRNA was amplified using the primers 338F (5′-ACT CCT ACG GGG AGG CAG CAG-3′) and 806R (5′-GGA CTA CHV GGG TWT CTA AT-3′) to investigate the bacterial communities. The PCR amplifications of the 16S ribosomal RNA (rRNA) gene and library preparation were performed by LC-Bio Technologies Co., Ltd. (Hangzhou, China). The libraries were sequenced either on 300PE MiSeq runs and one library was sequenced with both protocols using the standard Illumina sequencing primers, eliminating the need for a third (or fourth) index read.

### 2.4 Sequence processing and statistical analysis

Paired-end reads were merged using the software tool FLASH. Under specific filtering conditions, FQTRIM (v. 0.94) was used to filter the original tags to obtain high-quality clean tags. Chimeric sequences were filtered using Vsearch (v. 2.3.4). Sequences with similarities of ≥97% were assigned by Vsearch (v. 2.3.4) to the same operational taxonomic unit (OTU). Four indices were calculated to evaluate the α-diversity of each sample: Chao1, Observed species, Shannon’s index, and Simpson’s index. These indexes were calculated using QIIME (v. 1.8.0). Constrained principal component analysis (PCoA) of Bray-Curtis distances was used to assess differences in species complexity among samples. Significance of the PCoA was estimated using ADONIS. Core differential flora at the family level, using 10-fold cross-validation, was implemented with the rfcv () function in R with the package RandomForest. The linear discriminant analysis (LDA) effect size (LEfSe) was used to compare the abundance of all detected bacterial taxa among samples. The Kruskal–Wallis test was used to adjust for multiple testing (*p* < 0.05) and for the effect size analysis (LDA score of >3.5). The software PICRUSt2 was used for functional gene annotation of the 16S rDNA amplicon sequences (Douglas et al., 2019). Pathways were predicted using the KEGG database.

## 3 Results

### 3.1 Metadata and sequencing

**FIGURE 1:**
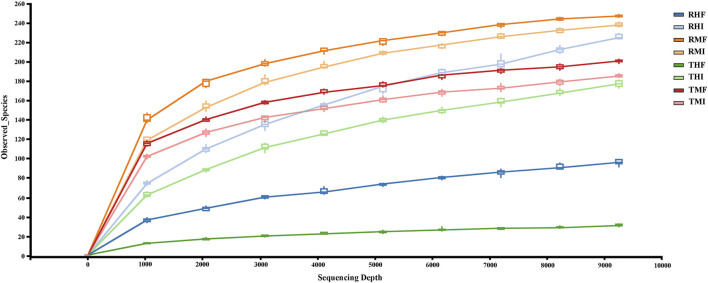
Rarefaction curves (Sobs) and estimators with different samples. Each rarefaction curve represents a sequencing group. The color of each curve corresponds to each intestinal sample group. RMI = IPRS, midgut, D7; RMF = IPRS, midgut, D90; RHI = IPRS, hindgut, D7; RHF = IPRS, hindgut, D90; TMI = PCS, midgut, D7; TMF = PCS, midgut, D90; THI = PCS, hindgut, D7; THF = PCS, hindgut, D90.

A total of 80 fish gut samples (40 midgut, 40 hindgut) were collected from juvenile GIFT for sequencing; 2,321,667 reads were assigned to 2,322 prokaryotic operational taxonomic units (OTUs) at 97% sequence similarity. In the rarefaction curves of Observed species (Figure 1), the end of the curve was flattened. Therefore, we concluded that the collected sequencing data was reasonable for our analyses.

### 3.2 Culture-mode effects on α and β-diversity of gut microbiota

**FIGURE 2:**
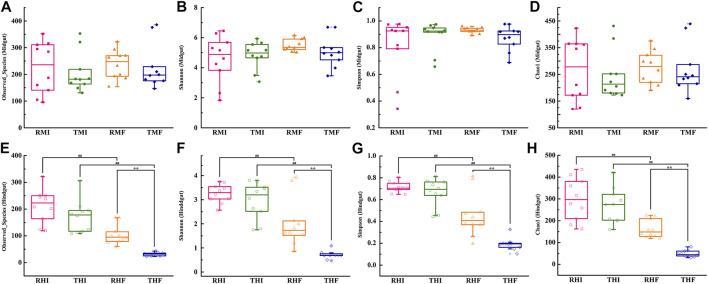
Alpha-diversity measurements of genetically improved farmed tilapia (GIFT) gut microbiota. **(A)** Observed species (midgut), **(B)** Shannon’s index (midgut), **(C)** Simpson’s index (midgut), **(D)** Chao1 index (midgut), **(E)** Observed species (hindgut), **(F)** Shannon’s index (hindgut), **(G)** Simpson’s index (hindgut), and **(H)** Chao1 index (hindgut). The symbols ## and ** indicate significant differences in abundance between two groups (Mann–Whitney *U*-test; **p < 0.01). For abbreviations, see caption to Figure 1.

The richness and diversity of bacterial species in intestinal flora were investigated by determining the values of the Observed species index (Figures 2A,E), Chao1 index (Figures 2B,F), Shannon’s index (Figures 2C,G), and Simpson’s index (Figures 2D,H). Between the IPRS and PCS culture modes, there was no significant difference in the α**-**diversity in midgut as of D7 and D90 (Figures 2A–D). Compared with on D7, the α-diversity of hindgut had decreased significantly by D90. At the same time, the decline in THF (PCS, hindgut, D90) was significantly greater than that in RHF (IPRS, hindgut, D90).

**FIGURE 3:**
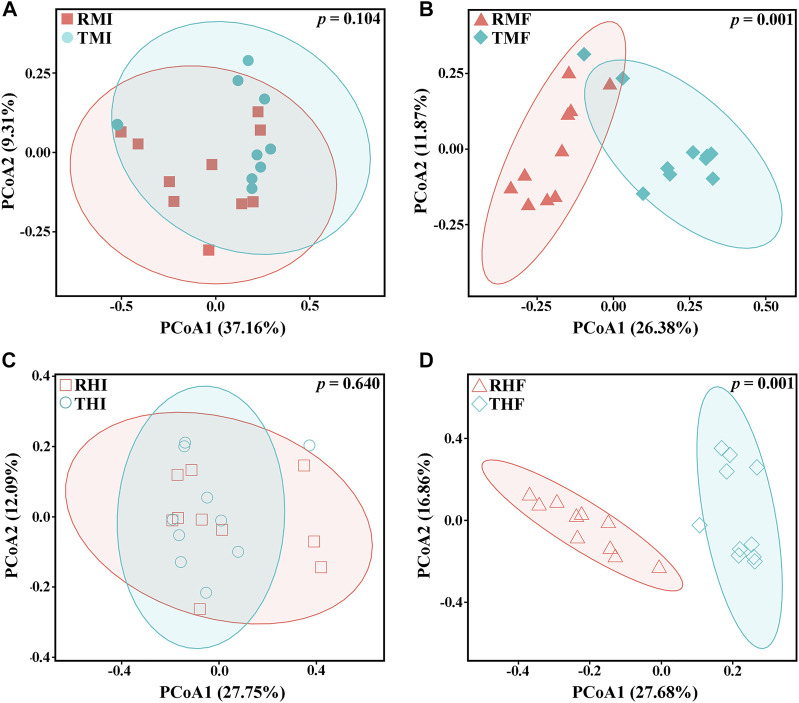
Plots of principal coordinates analysis (PCoA) of the gut microbiota of genetically improved farmed tilapia, based on unweighted UniFrac distance matrices. **(A)** RMI vs. TMI, **(B)** RMF vs. TMF, **(C)** RHI vs. THI, and **(D)** RHF vs. THF (for abbreviations, see caption to Figure 1). Each point represents a sample. Significance of the data was estimated using ANOSIM.

We also evaluated β-diversity of the gut bacteria to quantify differences in microbial community composition between the IPRS and PCS. Principal coordinate analysis (PCoA) based on unweighted (Figure 3) and weighted (Supplementary Figure S1) single fractal distance matrices was used to study the relationship between samples according to intestinal microbial community structure. In the PCoA of Bray–Curtis distance from samples, the midgut or hindgut samples clustered together on D7 and D90 (Figures 3A,C). As the experiment progressed, the samples between IPRS and PCS were gradually separated at D90 (Figures 3B,D), indicating that different culture modes were the main factor affecting the composition of the intestinal microbiota in the GIFT.

### 3.3 Culture-mode effects on dominant phyla of gut bacteria

**FIGURE 4:**
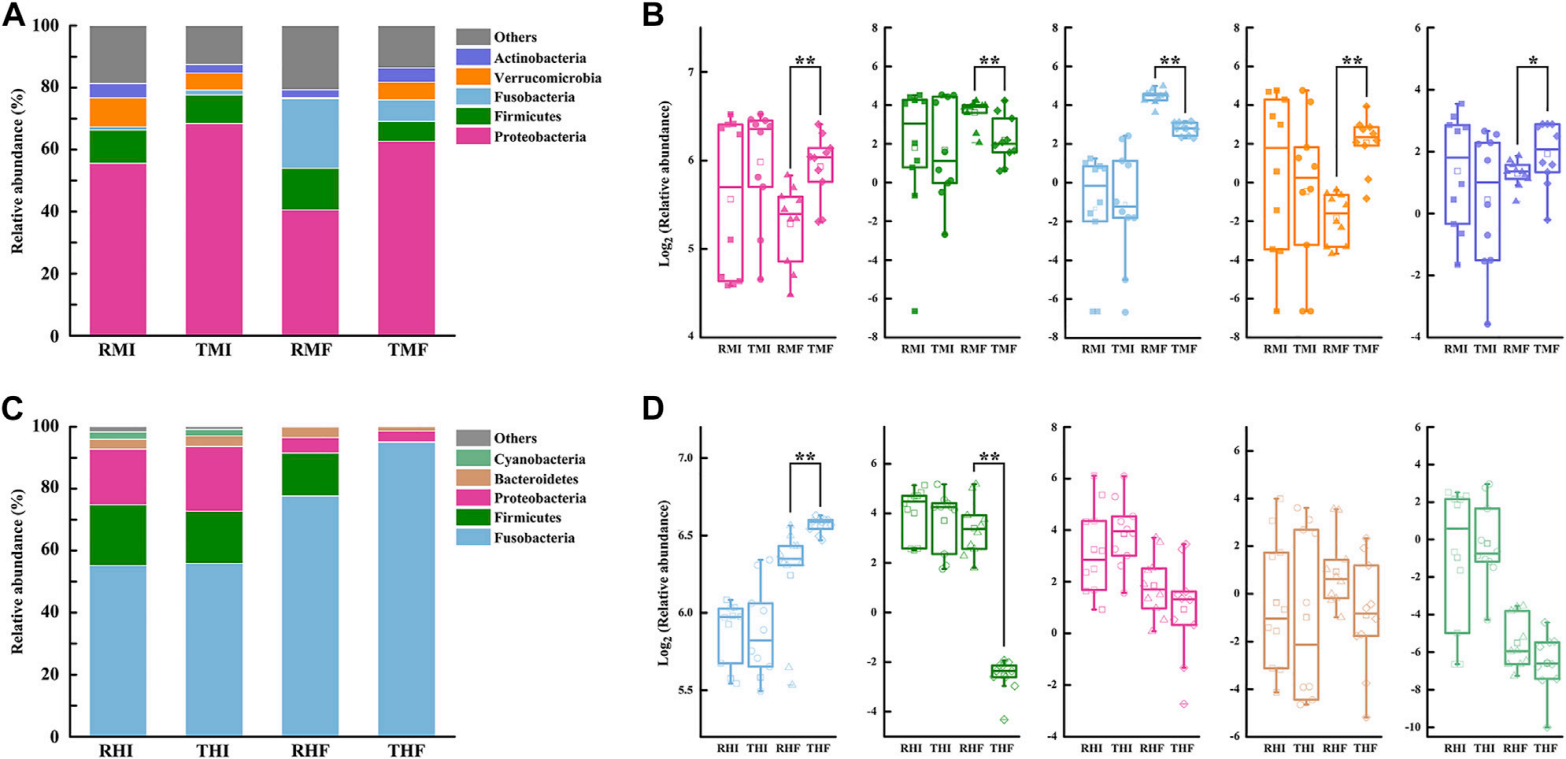
Histograms of relative abundance of intestinal bacteria in genetically improved farmed tilapia (GIFT), for predominant taxa identified at the phylum level, in **(A)** midgut and **(C)** hindgut (each bar represents relative abundance in each sample; only the five-most-abundant taxa are shown). Boxplots representing abundance of bacterial phyla in GIFT **(B)** midgut and **(D)** hindgut (lines in boxes represent medians of relative abundance). Significant difference in abundance between dietary groups are marked as *p < 0.05 or **p < 0.01 (Mann–Whitney *U*-test).

We further analyzed the composition of intestinal flora at the phylum level (Figure 4). Relative abundance of the five-richest OTUs in the intestinal microflora is depicted by a cumulative column chart (Figures 4A,C). At the phylum level, the composition of dominant bacteria had certain commonalities, with most sequences of 16S rRNA assigned to Proteobacteria, Firmicutes, and Fusobacteria. At the same time, the dominant bacteria showed specificity in the midgut and hindgut: the second-most-dominant bacteria phyla in the midgut were Verrucomicrobia and Actinobacteria, while the second-most-dominant in the hindgut were Bacteroidetes and Cyanobacteria. The relative abundances of the three most-dominant bacteria also differed significantly. The Proteobacteria dominated in the midgut, and its average relative abundance reached 56.81%; the average relative abundance of Firmicutes and Fusobacteria in midgut was 9.94 and 8.00%, respectively. In hindgut, the average relative abundance of Fusobacteria was as high as 70.91%, whereas the relative abundance of Firmicutes and Proteobacteria reached 12.66 and 11.80%, respectively.

Through the difference analysis, we found that there was no significant difference in the relative abundances of intestinal flora in fish under the different culture modes, whether in the midgut or hindgut, on D7. On D90, compared with the PCS midgut samples, the IPRS samples had more Firmicutes (13.44 vs. 6.37%) and Fusobacteria (22.42 vs. 6.95%) but less Proteobacteria (40.53 vs. 62.72%), Verrucomicrobia (0.38 vs. 5.72%), and Actinobacteria (2.53 vs. 4.62%) (Figure 4B). Only two bacteria phyla were represented in the hindgut; of these, the IPRS samples had a higher relative abundance of Firmicutes (13.93 vs. 0.19%), while the PCS samples had a higher relative abundance of Fusobacteria (94.92 vs. 77.62%) (Figure 4D).

### 3.4 Culture-mode effects on core gut bacterial taxa

**FIGURE 5:**
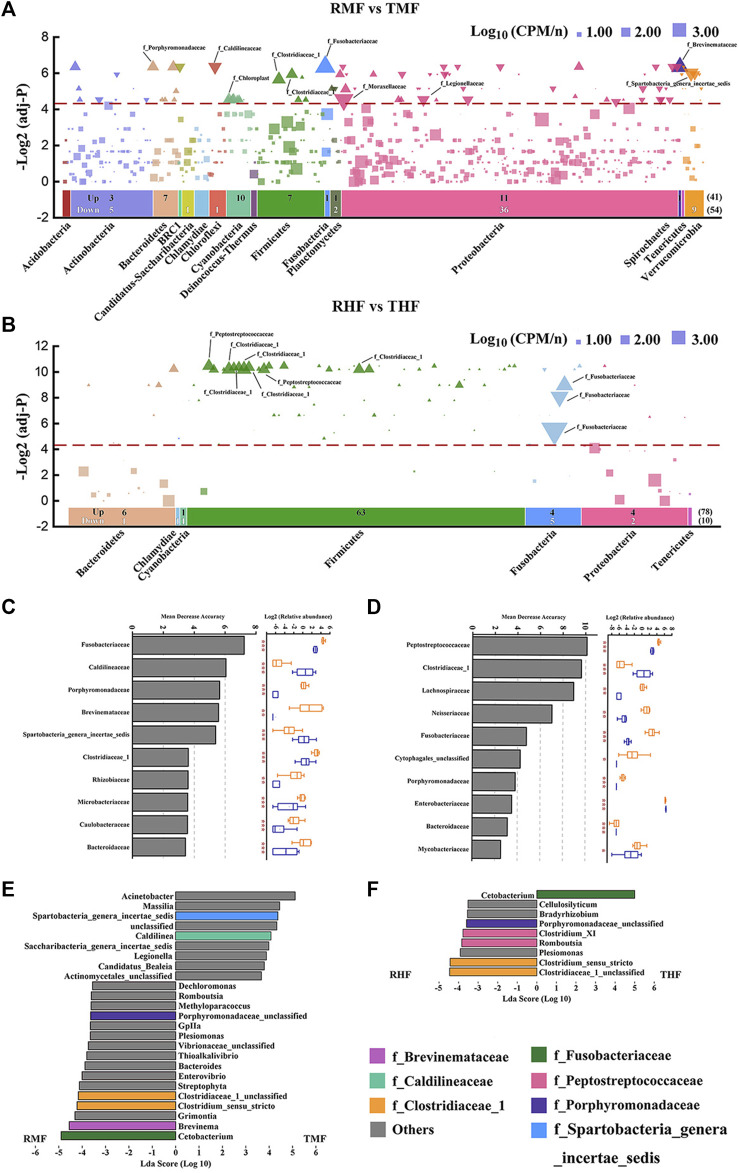
Differentially abundant gut bacterial taxa in genetically improved farmed tilapia (GIFT) between culture modes IPRS and PCS. Manhattan plots showing enriched OTUs in **(A)** midgut and **(B)** hindgut. No significant abundance of OTUs is depicted as a block; OTUs that are significantly enriched in IPRS are depicted as an upward triangle (▲); OTUs that are significantly enriched in PCS are depicted as a downward triangle (▼). Dashed lines correspond to the false discovery rate-corrected *p*-value threshold of significance (α = 0.05). The color of each dot represents the different taxonomic affiliation of the OTUs (phylum level), and the size corresponds to their relative abundance in the respective samples. Up: the amount of IPRS-enriched OUTs in different phylum; Down: the number of PCS-enriched OUTs in different phylum. The bacterial family of the 10 OTUs with the highest relative abundance are marked in the figure. The top-10 biomarker bacterial genera were identified by applying RandomForest regression of their relative abundances between IPRS and PCS in **(C)** midgut and **(D)** hindgut (Mann–Whitney *U*-test; *p < 0.05, **p < 0.01, ***p < 0.001). Linear discriminant analysis effect size (LEfSe) comparing abundance of bacterial genera between the IPRS and PCS conditions, in **(E)** midgut and **(F)** hindgut. The taxa shown in the histograms were determined to differ significantly in abundance between diets by Kruskal–Wallis test (*p* < 0.05) and have an LDA score of >3.5. Bacterial taxa associated with positive LDA scores (right) were overrepresented in PCS GIFT, and those with negative scores (left) were overrepresented in IPRS GIFT. The color of each bar represents the different taxonomic affiliation of the OTUs (family level).

We determined bacterial community shifts by arranging the OTUs according to their taxonomy and then displaying their enrichment in each the IPRS- and PCS-reared fish. The results revealed unexpectedly nuanced taxonomic alterations underlying the community shifts between midgut and hindgut (Figures 5A,B). The two panels in Figure 5 respectively filter the analysis results under the condition that the frequency of OTUs in group IPRS or PCS was >0.5. The number of OTUs screened in the midgut (691) was significantly higher than that in the hindgut (122). In contrast, the numbers of OTUs with significant differences between the midgut and hindgut were close (95 vs. 88). Notably, the number of OTUs enriched in the RMF (IPRS, midgut, D90) or TMF (PCS, midgut, D90) was similar (41 vs. 54) (Figure 5A), and the number of OTUs enriched in the RHF was much higher than that of THF (78 vs. 10) (Figure 5B). IPRS midgut-enriched OTUs belonged to the phyla Proteobacteria (11) and Cyanobacteria (10); PCS midgut-enriched OTUs belonged to the phyla Proteobacteria (36) and Verrucomicrobia (9). Whereas IPRS hindgut-enriched OTUs belonged to the phyla Firmicutes (63), PCS hindgut failed to enrich any member of the phyla Firmicutes (false discovery rate-corrected *p*-values, α = 0.05).

A random forest model was used to distinguish those gut bacterial families that could discriminate the microbial communities in the IPRS and PCS samples (Figures 5C,D). Families were ranked by their importance value (top-10). Manhattan plots reveal a total of seven core taxa screened at the family level: Fusobacteriaceae, Clostridiaceae 1, Peptostreptococcaceae, Brevinemataceae, Spartobacteria genera incertae sedis, Caldilineaceae, and Porphyromonadaceae. Bacteria at the genus level with an LDA value of >3.5 were screened by LEfSe (Figures 5E,F). Combining these three methods, four core genera were screened out, both in the midgut and hindgut: *Cetobacterium*, *Clostridium* sensu stricto, Clostridiaceae 1 unclassified, and Porphyromonadaceae unclassified.

### 3.5 Culture-mode effects on gut bacteria functions

**FIGURE 6:**
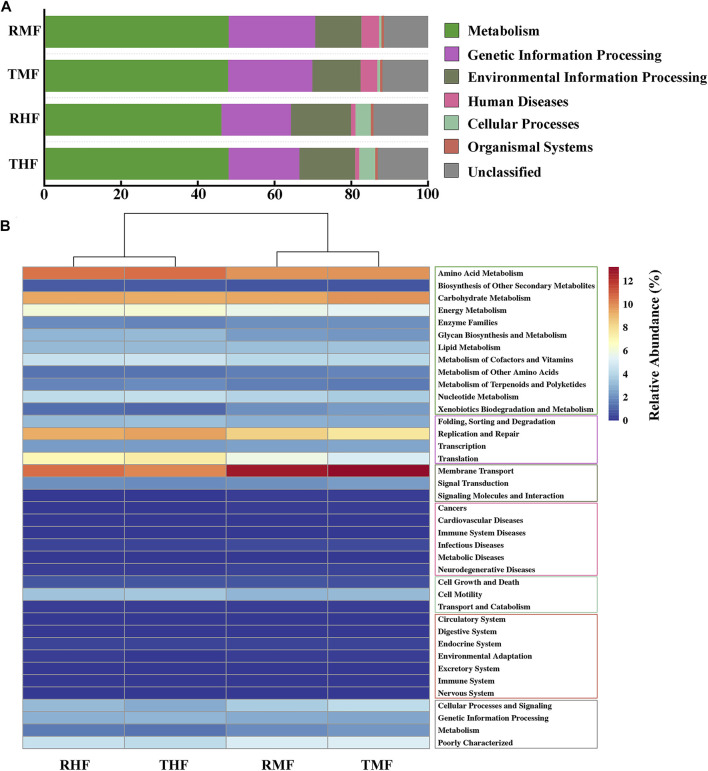
**(A)** Relative abundance of KEEG pathways (level 1). **(B)** Heatmap of functionally predicted KEEG pathways (level 2). The color represents the different KEEG pathways (level 1).

To compare the functional potential of the gut bacteria, functional abundance was predicted based on the OTUs using PICRUSt2. The predicted pathways were significantly enriched in the metabolism class at level 1 (Figure 6A), and the relative abundances in midgut and hindgut were 47.23 and 48.10%, respectively; this was followed by genetic information processing (21.97 vs. 22.52%) and environmental information processing (12.57 vs. 12.12%). In a total of 39 pathways at level 2 (Figure 6B), the pathways for amino acid metabolism, carbohydrate metabolism, and other metabolic pathways displayed high proportions. Other pathways with relatively high relative abundance were membrane transport and replication and repair.

## 4 Discussion

Rearing fish in IPRS units combines several biological, chemical, and physical intensification elements into a single, integrated system that can prove to be more controllable and efficient than traditional pond culture (Brown et al., 2011). We were genuinely concerned about the health of fish (via their intestinal flora) under the high-density culture mode of IPRS; therefore, the present study aimed to describe compositional and functional differences of the gut microbiome of GIFT reared under culture modes IPRS or PCS, by investigating their responses to a 90-days culture experiment.

### 4.1 Changes of intestinal microbial community structure under IPRS and PCS

Our previous studies on fish intestinal microorganisms clarified that the composition of intestinal microorganisms will be significantly affected by the feed nutrients (Zhu et al., 2020a; Zhu et al., 2020b). The present study shows that the rearing method can also significantly affect the composition of GIFT intestinal microbial community. The PCoA showed that the microbial community structure of tilapia intestinal midgut and hindgut changed significantly as the 90-days experiment progressed.

First, we focused on the most abundant gut microbial phyla. The microbial composition of the gut generally remains stable in the same species. Fan et al. (2017) characterized microbial communities in the gut of intensively cultured GIFT during the peak breeding period, and found that the dominant bacterial phyla were Proteobacteria, Actinobacteria, and Firmicutes. Similarly, our previous research (Zhu et al., 2020b) found that the dominant phyla in the gut of GIFT were Fusobacteria, Bacteroidetes, Proteobacteria, and Firmicutes. In the intestinal samples from the current experiment, the most dominant bacteria phyla were Proteobacteria, Fusobacteria and Firmicutes. Qin et al. (2010) reported that Bacteroidetes and Firmicutes are the dominant bacteria in the intestinal tract of most vertebrates, constituting over 90% of the known phylogenetic categories. However, we found that the relative abundance of Bacteroidetes and Firmicutes was only around 10%. Among the 16 bacterial phyla identified in the guts of juvenile GIFT in our study, Proteobacteria and Fusobacteria were the most abundant in the midgut and hindgut, respectively. Proteobacteria exists widely in aquatic environments (Ley et al., 2008). The life cycles and growth environments of aquatic animals are more diverse than those of land animals; accordingly, the intestinal microbiota would be more diverse in aquatic organisms than in terrestrial animals. The core gut bacteria of fish is, in general, more influenced by the environment, since aquatic environments are likewise rich in bacteria (Tsuchiya et al., 2010; Banerjee and Ray, 2017). This is likely an important factor for the difference between the relative abundance of gut bacteria in GIFT and higher vertebrates. Whether in the midgut of IPRS or PCS fish, the relative abundance of Proteobacteria decreased gradually with the progress of the experiment, while the relative abundance of Fusobacteria increased. In the hindgut, the relative abundance of Fusobacteria significantly increased, and the relative abundance of both Proteobacteria and Firmicutes decreased. Proteobacteria is the largest branch of prokaryotes, accounting for the vast majority of known Gram-negative bacteria. Since many Proteobacteria are opportunistic bacteria, this can increase the chance of diseases. One study found that excessive Proteobacteria may be a sign of dysbiosis of intestinal microorganisms (Shin et al., 2015). In the midgut, the relative abundance of Proteobacteria was high across the whole experimental period. The relative abundance of Proteobacteria was significantly higher in PCS samples than in IPRS samples on D90.

Second, we focused on the change of gut microbiome α-diversity. The richness and diversity estimators representing the integral status of the gut microbiome appear to be ideal predictors in analyzing the structure of the microbiota. Numerous studies proved that the number of flora in different sections of the digestive tract in a variety of fish showed a gradual upward trend from front to back, and the α-diversity of the intestinal flora was correspondingly higher (Molinari et al., 2003; Navarrete et al., 2009; Ye et al., 2014). In this study, the diversity index of GIFT midgut was higher than that of hindgut, which is contrary to the results of most fish studies, though similar to a report for pond frog *Rana rugulosa* (Liu et al., 2020). Whether reared under the IPRS or PCS conditions, 90-days artificial intensive culture reduced the intestinal flora diversity of GIFT. Microbial diversity is closely related to the health of animals, and species diversity promotes stability and performance (Shin et al., 2015; Liu et al., 2018). Compared with animals fed under normal conditions, those fed under aseptic conditions were more susceptible to infection and their gastrointestinal immune function was weaker (Ley et al., 2006). Therefore, higher diversity may be an important indicator of healthy microflora. The decrease in diversity may have destabilized the microflora, and weakened the host’s ability to combat disease. However, the IPRS culture mode had less impact on α-diversity when compared with the PCS.

Different parts of the intestine of higher animals usually assume different physiological functions. Differences in microbiota composition and diversity in different intestinal segments have been reported in higher animals such as mice (Gu et al., 2013), pigs (Looft et al., 2014), and chickens (Choi et al., 2015). Studies of fish (lower vertebrates) have shown that various digestive enzymes, hydrolases, and oxidase in different parts of the intestine are differently distributed, which reveals differentiation of the digestion, absorption, and immune defense functions in different parts of the intestine (Wang et al., 2017). Roeselers et al. (2011) reported that the abundance of Fusobacteria in the intestine varied greatly between fish in different locations. Our study confirmed significant differences in the relative abundance of Fusobacteria in different intestinal segments in farmed tilapia. However, there may be differences in the responses of intestinal microorganisms in different segments of the fish intestine under the same culture mode. Although the aquaculture mode affects the composition of the dominant intestinal bacteria, the change in the direction of the relative abundances of the dominant phyla may be the opposite in different intestinal segments. This was most evident in the α-diversity of the gut microbiome in our experiment. Between the IPRS and PCS culture modes, there was no significant difference in the α-diversity in the midgut of GIFT at D90, whereas the α-diversity in the hindgut had decreased significantly.

Intestinal microflora is a complex group of microorganisms that inhabit the gastrointestinal tract of fish. Microflora are closely related to many aspects of normal host physiology, from nutritional status to behavior and the stress response. The density of IPRS was more than 10 times that of PCS, however, various problems emerge with intensive fish culture methods, especially crowding stress and increased susceptibility to disease, which will ultimately influence the growth performance, welfare, and profitability of the farmed fish. The continuous push of water through the units is a feature of IPRS, so the dissolved oxygen of water is more sufficient, and the waste is removed in time. Integrate the date obtained from our 90days-feeding experiments, to summarize, we believe that a more diverse gut microbiome with a lower relative abundance of Proteobacteria is more conducive to good growth and disease resistance in IPRS-reared GIFT.

### 4.2 Biomarker taxa correlated with IPRS and PCS

To gain insight into the microbial basis of fish gut changes based on the culture mode, it is necessary to determine which microorganisms can be attributed to the observed changes. However, depth analysis can be difficult with complex multi-dimensional microbiota data. Only 95 OTUs among a total of 2,322 OTUs in our midgut dataset represent the most abundant taxa, with significant differences evident between the different culture modes. The number of OTUs with significant differences in the hindgut was 88. However, it is noteworthy that the top-10 OTUs (relative abundance) of the midgut belonged to nine families, whereas the OTUs of the hindgut were concentrated in three families. Fusobacteriaceae was the only family enriched in PCS hindgut. We further identified biomarker families correlating with culture mode by using a RandomForest model to explore the complex nonlinear interdependence between microorganisms. According to the above two analysis methods, seven core taxa at the family level were screened out. The LEfSe analysis at a 3.5-threshold level was used to compare the relative abundances of the bacterial taxa at the genus level; the results show the core taxa belonged to seven core families. It is noteworthy that in both the midgut and hindgut IPRS samples, the enriched genera were *Clostridium* sensu stricto, Clostridiaceae 1 unclassified, and Porphyromonadaceae unclassified. Interestingly, the genus *Cetobacterium* within the family Fusobacteriaceae was significantly enriched in IPRS midgut samples, yet also enriched in PCS hindgut samples. *Cetobacterium* was the most dominant bacterial genus in the GIFT hindgut. *Cetobacterium* is part of the core microbiome in several fishes (Larsen et al., 2014; Sun and Xu, 2021) and it was mainly distributed in the hindgut of the GIFT. We speculate that this is because *Cetobacterium* is anaerobic in nature, and hence the absolute anaerobic environment of the hindgut is more suitable for its colonization. Gut microbiota with a greater abundance of acetate-producing *Cetobacterium* improves carbohydrate utilization, and thereby improves glucose homeostasis in fish (Wang et al., 2021). The capacity of carbohydrate digestion and metabolism is generally poor in fishes, but a high abundance of *Cetobacterium* in the intestine can effectively improve this situation. These bacteria also have vitamin-producing capacity by fermenting carbohydrates and peptides to produce large quantities of vitamin B-12, which has been proven to promote intestinal health in many fish species (Ramírez et al., 2018).While *Cetobacterium* was enriched in hindgut of PCS samples, it was enriched in midgut of IPRS samples. During the culture period, the intestinal flora of tilapia in different culture systems develop under different patterns of succession on temporal and spatial scales. The genus *Clostridium* sensu stricto and Clostridiaceae 1 unclassified within the family Clostridiaceae 1 were significantly enriched in both IPRS midgut and hindgut. The main acetate-producing bacteria are *Clostridium* species, which also produce butyrate and propionate (Xu et al., 2020). These short-chain fatty acids (SCFAs) can be absorbed by the body as a nutrient and contribute to the host’s energy metabolism (LeBlanc et al., 2017). SCFAs also represent key modulators of physical performance. SCFAs can reduce pH levels and maintain the intestinal micro ecosystem, and mediate the signal of G-protein-coupled receptor 41/43 (GPCR-41/43), and so affect glucose and lipid metabolism (Duncan et al., 2010). Training and regular exercise have been associated with increased fecal SCFA contents in humans (Barton et al., 2018), and specific SCFAs have been associated with improved physical performance in animal studies (Okamoto et al., 2019). A continuous push of water through the unit is a characteristic of the IPRS, consequently the cultured fish remain in motion. This may explain why *Clostridium* sensu stricto was enriched in both midgut and hindgut of the IPRS fish. Furthermore, the flow velocity in the IPRS has a great influence on water quality, which benefits fish growth, and an appropriate flow velocity is a necessary condition for ensuring the welfare of farmed fish.

### 4.3 Functional features of gut bacteria shaped by IPRS and PCS

The analysis with PICRUSt2 revealed that the IPRS and PCS were similar in their effects on gut microbial metabolic functions. The intestinal microflora had similar abundance distribution characteristics of functional pathways. The metabolic activity of intestinal bacteria is especially active, and the predicted pathways were significantly enriched in the metabolism class at level 1 (Figure 6A), with relative abundance around 50%**.** We found that the intestinal microorganisms mainly had metabolic functions, but they also played unique functions in genetic information processing and environment information processing. The difference between different intestinal segments was mainly in human diseases and cellular processes. It is worth noting that in this functional prediction, disease-related microorganisms also account for a certain proportion, which revealed the complex relationship between intestinal microorganisms and host immunity. At the level-2 pathway, the relative abundance of functions related to amino acids, carbohydrates, energy, cofactors, and vitamin metabolism were high (>4%). As mentioned above, certain intestinal flora can effectively improve the digestion and metabolism of carbohydrates in fish. The survival of bacteria in the digestive tract depend on the host’s diet and thus on nutrition-derived proteins and amino acids. At the same time, the metabolic activities of intestinal bacterial groups may also affect the amino acid homeostasis and health status of the host (Neis et al., 2015). Some fish intestinal microflora also play an important role in regulating the metabolism, transport, and storage of lipids (Semova et al., 2012). Nayak (2010) found that *Clostridium* and *Cetobacterium* in fish intestinal flora were closely related to the synthesis of vitamin B12. We speculate that diversity functions of the intestinal flora would play an important role in metabolism and help to maintain a stable microbiota structure. The cluster analysis also showed that the effect of different intestinal segments on the function of intestinal flora was greater than that of different culture modes. Owing to the differences of physical and chemical properties in different parts of the digestive tract, the number and structure of intestinal flora were vastly different. This result corresponds to the difference in microbial composition between the midgut and hindgut.

## 5 Conclusion

The present study analyzed the profiles of microbial communities in GIFT under two different culture modes, and describes the characteristic composition of gut bacteria between IPRS- and PCS-reared fish. Furthermore, we compared the functional potential of bacteria, with functional abundance predicted based on the OTUs, using PICRUSt2 software. During the culture period, the intestinal flora of the tilapia in different culture systems underwent different succession patterns on temporal and spatial scales. Whether reared under the IPRS or PCS conditions, 90-days intensive culture reduced the intestinal flora diversity of the GIFT. Moreover, this study showed that the IPRS GIFT intestinal microorganisms with higher diversity and lower relative abundance of Proteobacteria were more conducive to their growth and resistance to disease. The intestinal microflora had similar abundance distribution characteristics of functional pathways. The metabolic activity of intestinal bacteria is especially active, and the effect of different intestinal segments on the functions of the intestinal bacteria was greater than that of different culture modes. These results are encouraging and may indicate that the IPRS is a promising ecological production strategy that more efficient to remain profitable and environmentally sustainable for culturing tilapia in China.

## Data Availability

The datasets presented in this study can be found in online repositories. The names of the repository/repositories and accession number(s) can be found below: https://www.ncbi.nlm.nih.gov/bioproject/PRJNA839702.
