# Supplementary material for: Multi-kingdom microbiota and functions changes associated with culture mode in genetically improved farmed tilapia (Oreochromis niloticus)
Source: Front Physiol. 2022 Sep 12;13:974398. doi: 10.3389/fphys.2022.974398 (PMC9510917; doi:10.3389/fphys.2022.974398)
Supplement: Supplementary file 1 [file Table1.DOCX]

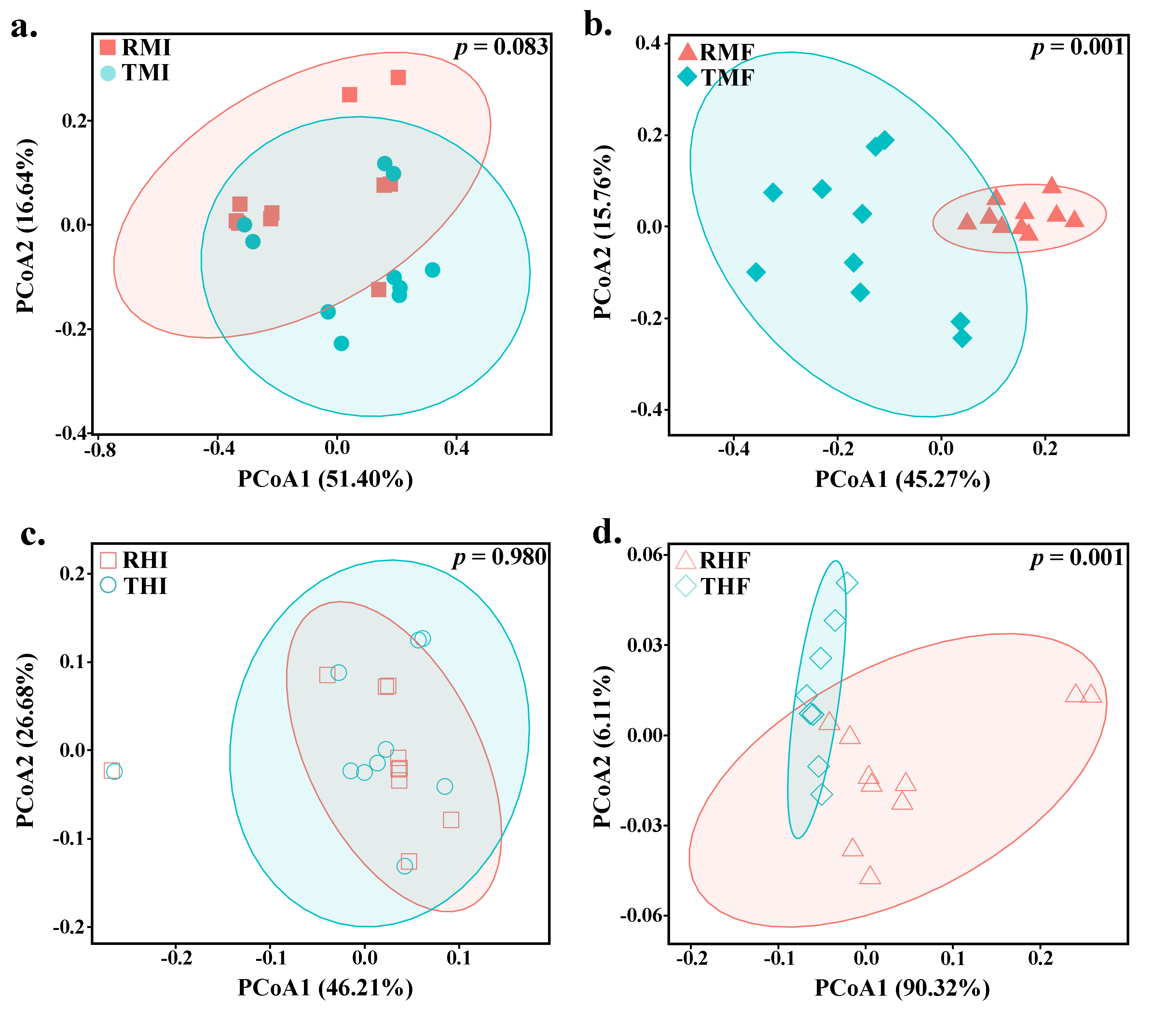


Fig. S1. Plots of principal coordinates analysis (PCoA) of the gut microbiota of genetically improved farmed tilapia, based on weighted UniFrac distance matrices. a) RMI vs TMI, b) RMF vs TMF, c) RHI vs THI, and d) RHF vs THF. Each point represents a sample. Significance of the data was estimated using ANOSIM.
